# Evaluating SnapshotNIR for Tissue Oxygenation Measurement Across Skin Types After Mastectomy

**DOI:** 10.3390/bioengineering12080892

**Published:** 2025-08-21

**Authors:** Saif Badran, Sara Saffari, William R. Moritz, Gary B. Skolnick, Amanda M. Westman, Mitchell A. Pet, Justin M. Sacks

**Affiliations:** Division of Plastic and Reconstructive Surgery, Department of Surgery, Washington University School of Medicine, St. Louis, MO 63110, USAgskolnic@wustl.edu (G.B.S.);

**Keywords:** Fitzpatrick, flap monitoring, mastectomy, necrosis, near-infrared spectroscopy, snapshot, personalized medicine, tissue oxygenation

## Abstract

Accurate monitoring of mastectomy skin flap (MSF) perfusion is critical, especially in patients with darker skin pigmentation at higher risk of misdiagnosed tissue ischemia. Near-infrared spectroscopy (NIRS) devices, such as SnapshotNIR, offer real-time tissue oxygen saturation measurements (StO_2_), but their accuracy across skin pigmentation levels remains unexplored. This quasi-experimental study included 33 patients undergoing mastectomy. MSF edge ΔStO_2_, defined as preoperative minus postoperative StO_2,_ was measured using SnapshotNIR device (Kent Imaging, Calgary, AB, Canada) pre- and post-mastectomy. By definition, a positive ΔStO_2_ indicates a decrease in tissue oxygenation, while a negative ΔStO_2_ indicates an increase relative to baseline. ΔStO_2_ was analyzed against Fitzpatrick scores to assess skin pigmentation impact on measurement accuracy. ΔStO_2_ (mean ± SD) progressively decreased with increasing Fitzpatrick score: 14.0 ± 22.98 for score 1, 6.87 ± 17.45 for score 2, −3.13 ± 6.89 for score 3, and −40.75 ± 22.27 for score 5, indicating a shift from positive to negative O_2_ change. Fitzpatrick scores significantly correlated with ΔStO_2_ (ρ = −0.392, *p* = 0.016). ANOVA confirmed differences (*p* = 0.008), with Tukey’s post hoc testing showing significant differences between Fitzpatrick scores 1 and 5 (*p* = 0.022), and 2 and 5 (*p* = 0.006). SnapshotNIR technology demonstrated measurable sensitivity for detecting changes in StO_2_ and predicting ischemia; however, NIRS-based devices may overestimate oxygenation in darker skin pigmentation, highlighting a need for device calibration to improve accuracy across skin tones.

## 1. Introduction

Breast cancer remains the most common cancer among women worldwide [1], and surgical management often involves partial or complete resection of breast tissue, either for therapeutic or prophylactic purposes. Skin-sparing and nipple-sparing mastectomies preserve the overlying skin envelope; however, these techniques compromise the skin’s primary blood supply from underlying vessels, rendering it reliant solely upon the subdermal vascular network, resulting in reduced perfusion. This hypoperfusion status contributes to the risk of mastectomy skin flap (MSF) necrosis and related complications, reported in up to 30% of cases. This is of particular importance following immediate implant-based alloplastic reconstruction [2,3], where the implant or expansion process exerts tension forces over the MSF and further reduces tissue perfusion [4].

In breast reconstruction, maintaining the viability of MSF is critical to prevent complications such as wound breakdown and implant exposure requiring explantation. Severe complications may also include infection, delay in adjuvant therapy, and reconstructive failure [2]. Several clinical factors significantly increase risk of MSF necrosis, including higher body mass index (BMI), prior surgical scars, greater mastectomy weight, history of smoking, radiation therapy, and previous breast surgeries [5]. However, the presence or absence of these risk factors does not adequately predict tissue variability in the individual patient, and clinical evaluation such as skin color, capillary refill time, and flap temperature remains the gold standard for assessing tissue perfusion [6,7,8,9]. Various adjuncts to clinical flap monitoring, such as Doppler ultrasound, implantable Dopplers, and hyperspectral imaging, have been implemented to improve the early detection of ischemia and reduce postoperative complications. Identifying these risks and employing advanced monitoring techniques supports better clinical decision-making, such as staging reconstruction procedures or enhancing postoperative surveillance [6,7,8,9].

Previous studies have demonstrated that individuals with darker skin pigmentation are more likely to experience misdiagnosed tissue ischemia by means of physical examination alone, emphasizing the need for accurate and objective assessment tools [10]. Among these devices, near-infrared spectroscopy (NIRS)-based transcutaneous tissue oximetry has emerged as a valuable tool in plastic surgery, enabling repetitive and non-invasive postoperative monitoring of cutaneous free flaps for early detection of vascular compromise [9,10,11,12,13,14,15,16]. This apparatus demonstrated superior sensitivity and specificity compared to available alternatives such as implantable Dopplers, with a reported reduction in total flap loss rates by up to 80%. However, their use is predominantly focused on the early postoperative period, and they are restricted to a single point measurement on the flap [13,17,18].

SnapshotNIR (Kent Imaging, Calgary, AB, Canada) is an FDA-approved, non-invasive, reflectance-based technology designed to measure tissue oxygen saturation (StO_2_) in superficial tissues. By utilizing multiple wavelengths of NIR light, SnapshotNIR quantifies the relative amounts of oxygenated and deoxygenated hemoglobin in the microcirculation, where oxygen exchange occurs. This system generates a tissue oxygenation map that can be used in medical decision-making for tracking and trending oxygenation, as well as evaluating tissue viability in wound care [4]. Racial bias has been reported in devices like pulse oximeters, which have been shown to increase the risk of undetected hypoxemia in individuals with darker skin pigmentation. This can potentially lead to inadequate patient triage and inappropriate adjustments of oxygen therapy [16,19,20]. Consequently, this study aimed to evaluate the efficacy of the SnapshotNIR device, which has demonstrated success in identifying spatial and temporal variations in tissue oxygenation during both alloplastic and autologous breast reconstruction. However, its performance across different skin pigmentation levels remains unknown, representing a critically important gap in knowledge [21]. The objective of this research was to assess the accuracy of the SnapshotNIR in monitoring changes in skin flap perfusion following mastectomy, and to determine how skin pigmentation, quantified using the Fitzpatrick scale, affects its reliability.

## 2. Materials and Methods

### 2.1. Study Design

This pilot study employed a prospective quasi-experimental (before-and-after) design in which each patient served as their own control, comparing pre- and post-mastectomy measurements to reduce inter-patient variability and strengthen internal validity despite the absence of randomization. This study was approved by the Washington University Human Research and Protection Office (approval no. 202103145).

### 2.2. Enrollment

Adult females undergoing nipple- or skin-sparing mastectomy with immediate reconstruction using tissue expanders or implants (alloplastic reconstruction) or two-stage free flap reconstruction (autologous reconstruction) at the Washington University in St. Louis (St. Louis, MO, USA) were considered for eligibility. For bilateral cases, each breast was treated as an individual case study with the same patient demographics, as each breast has distinct vascular anatomy, and variations in surgical technique inherently result in unique perfusion outcomes. This approach does not violate independence in analysis, as each breast was analyzed separately to account for these individual differences. Patients were excluded if they declined consent or if SnapshotNIR data were incomplete; no exclusions were applied for comorbidities. Written informed consent, including consent for photography, was obtained from all participants.

### 2.3. Study Subjects

Preoperative imaging of the proposed skin incision sites was performed after the surgical team completed preoperative markings. The second set of images was taken immediately after completion of the mastectomy and prior to skin closure or any breast reconstruction. This ensured that measurements were obtained before implant placement or closure-related tension, thereby avoiding any confounding factors, including mechanical artifacts that could impact perfusion assessment. The surgeons and clinical team were blinded to the SnapshotNIR measurements to ensure that clinical decision-making and patient care were not influenced by the device readings. This blinding was necessary to independently validate the device’s accuracy and reliability in assessing tissue oxygenation. All imaging was performed by a designated research member who was not involved in clinical decision-making. Comprehensive clinical and demographic data collected included the following:

- Patient age;
- BMI;
- Skin phenotype;
- History of prior breast surgery;
- Smoking history;
- Relevant medical history;
- Use of adjuvant therapy for breast cancer (e.g., hormonal therapy, target, immunotherapy or any type of adjuvant therapy);
- Perioperative data.

The skin phenotype was recorded according to the Fitzpatrick classification (Table 1) [22].

### 2.4. Image Capture

Images were captured using the SnapshotNIR system (Kent Imaging Inc., Calgary, AB, Canada). Subjects were intraoperative normothermic and hemodynamically stable as per the standard of the anesthesia protocol. Ambient light was controlled during image capture under standard operating room lighting to maintain consistency and accuracy in the measurements. SnapshotNIR and other NIRS-based technologies are inherently less susceptible to interference from ambient visible light due to their use of longer wavelengths. Additionally, the device’s built-in processing algorithms correct for any minor interference patterns, ensuring that these effects do not influence perfusion analysis.

The SnapshotNIR device used does not require a calibration routine prior to use, as it is factory-calibrated and designed for immediate operation. The device was positioned parallel to the surgical site at a standardized distance of 30 cm, enabling the assessment of perfusion characteristics within a 15 cm × 20 cm field of view. The SnapshotNIR system simultaneously captures a digital color photograph and a spatially matched StO_2_ image, ensuring precise dataset alignment for analysis. During each use, it also generates an RGB image, a spatial map of pixel-level StO_2_ measurements, and hemoglobin reflectance images (oxyhemoglobin, deoxyhemoglobin, and total hemoglobin).

### 2.5. Image Analysis

**Figure 1:**
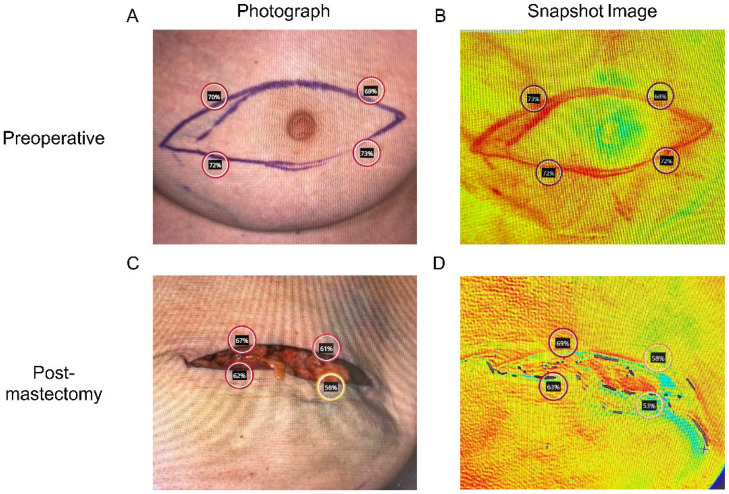
Tissue oxygenation saturation (StO_2_) measurement using Snapshot NIR technology (**A**,**B**) prior to, and (**C**,**D**) immediately following mastectomy.

A blinded reviewer selected areas from the color images for analysis. The first set of measurements was taken around the proposed mastectomy incision before the mastectomy (Figure 1A,B). The second set of measurements was taken at the corresponding sites of the first measurements, representing the MSF edges, which are areas with the highest ischemia (Figure 1C,D). Within each area of interest on the color image, StO_2_ measurements were obtained from three selected locations, of which the values were averaged to account for outliers. Areas of skin that were shadowed, reflective, or otherwise obscured were avoided. For patients undergoing bilateral mastectomy and breast reconstruction, each side was measured separately. The primary outcome was the StO_2_ drop (ΔStO_2_), defined as preoperative StO_2_ minus postoperative StO_2_, measured immediately after mastectomy and prior to skin closure or breast reconstruction (alloplastic or autologous). Positive ΔStO_2_ values indicate reduced tissue oxygenation, whereas negative values indicate increased oxygenation relative to baseline, a distinction critical for accurate interpretation of these findings.

### 2.6. Data Analysis

Descriptive statistics were calculated. Bivariate analysis assessed the relationships between ΔStO_2_ and other variables (e.g., patient demographics). Normality of continuous variable distributions was assessed using the Shapiro–Wilk test. Parametric testing including Student’s *t*-tests and Pearson’s correlations was applied to normally distributed variables. Levene’s test for homoscedasticity determined which form of the Student’s *t*-test was applied. Non-parametric testing including Mann–Whitney U and Spearman’s correlations was applied to the other continuous variables. The bivariate relationship of ΔStO_2_ and (ordinal-scale) Fitzpatrick scores was assessed using both Spearman’s correlation and one-way analysis of variance (ANOVA) with Tukey’s HSD post hoc testing. Spearman’s rank correlation was employed to assess associations with the ordinal Fitzpatrick scale, while one-way ANOVA was utilized to compare mean perfusion values across its categorized groups. A *p*-value threshold of 0.10 was used to assess normality, while statistical significance for all other analyses was set at *p* < 0.05. Analyses were performed using IBM SPSS Statistics for Windows, Version 29.0.0.0 and GraphPad Prism Version 10.1.1.

## 3. Results

### 3.1. Study Population, Measurements, and Associations with ΔStO_2_

A total of 33 subjects were included in the study from July 2022 to July 2023. Mean ΔStO_2_ was 2 ± 23 with a maximum drop of 46 and a maximum increase of 57. The mean age of subjects was 49 ± 12 years. Fitzpatrick skin scores were distributed as follows: 2 subjects (6.1%) with score 1 (Fitzpatrick type I: very fair); 24 subjects (72.7%) with score 2 (Fitzpatrick type II: fair); 2 subjects (6.1%) with score 3 (Fitzpatrick type III: average skin color); and 2 subjects (6.1%) with score 5 (Fitzpatrick type V: dark brown). No subjects had a Fitzpatrick score of 4 or 6 (Fitzpatrick type IV: light-brown skin; Fitzpatrick type VI: black skin), and scores were missing for 3 individuals (9.1%). Age and ΔStO_2_ were treated as normally distributed (Shapiro–Wilk: 0.103 ≤ *p* ≤ 0.490), while mean intraoperative vital sign measurements (temperature, systolic and diastolic blood pressure, and heart rate) were treated as non-normally distributed (0.010 ≤ *p* ≤ 0.077). Associations between binary clinical variables and ΔStO_2_ are summarized in Table 2. Smoking status, comorbidities, previous breast surgery, and postoperative complications were not significantly associated with ΔStO_2_ (*p* > 0.05 for all comparisons). Among adjuvant therapies, only hormonal therapy (6.1% of the participants) was significantly associated with ΔStO_2_ (mean ΔStO_2_: 37.9 ± 7.2 vs. −0.3 ± 21.5; *p* = 0.019). No significant differences in ΔStO_2_ were observed for patients receiving chemotherapy, targeted therapy, immunotherapy, or any adjuvant therapy combined (*p* > 0.05 for all comparisons). None of the correlations of vital signs (e.g., temperature, systolic blood pressure, diastolic blood pressure and heart rate) with ΔStO_2_ were found to be statistically significant (0.054 ≤ *p* ≤ 0.586).

### 3.2. Influence of Fitzpatrick Score on ΔStO_2_

**Figure 2:**
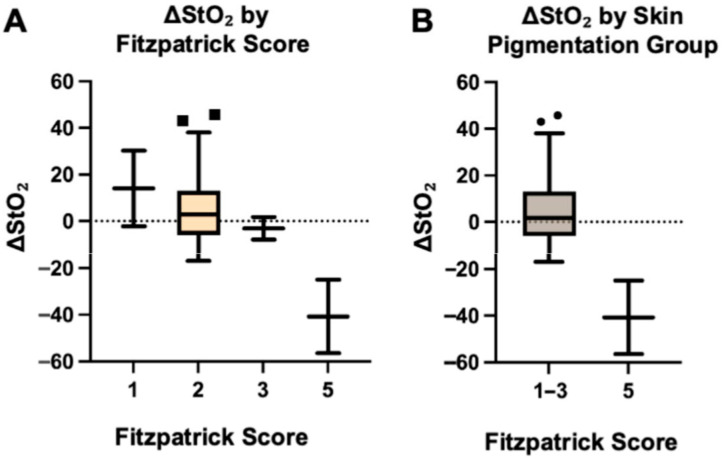
Change in tissue oxygenation (ΔStO_2_), by Fitzpatrick score (**A**) and skin pigmentation group (**B**).

ΔStO_2_ at the MSF edges, defined as preoperative StO_2_ minus postoperative StO_2_, was highest in subjects with Fitzpatrick score 1 (mean ± SD [95% confidence intervals (CI)]: 14.0 ± 22.98, CI: −192.5 to 220.5) and progressively decreased with increasing Fitzpatrick score: 6.87 ± 17.45 (CI: −0.5 to 14.2) for score 2; −3.13 ± 6.89 (CI: −65.1 to 58.8) for score 3; and −40.75 ± 22.27 for score 5 (CI: −240.9 to 159.4). This reflects a shift from positive to negative O_2_ drop (Figure 2A). A positive ΔStO_2_ indicates a perceived decrease in tissue oxygenation after mastectomy, while a negative value indicates an increase relative to preoperative baseline. Fitzpatrick score was significantly associated with ΔStO_2_ based on Spearman’s correlation (ρ = −0.392, *p* = 0.016), indicating an inverse relationship between skin pigmentation and oxygenation change, and confirmed using one-way ANOVA (*p* = 0.008). Post hoc comparisons revealed significant differences between Fitzpatrick scores 1 and 5 (*p* = 0.022) and 2 and 5 (*p* = 0.006). Comparison of lighter pigmented skin (Fitzpatrick scores 1–3) to darker pigmented skin (Fitzpatrick score 4–6) demonstrated significantly higher ΔStO_2_ in the lighter pigmented skin group (median 1.75 vs. −40.75; *p* = 0.005, Mann–Whitney U = 0, Figure 2B). The Hodges–Lehmann estimate for the difference in medians was −48.5.

The box plots show ΔStO_2_, calculated as preoperative StO_2_ minus postoperative StO_2_, stratified by Fitzpatrick skin type (1, 2, 3, and 5; Figure 2A). 

The box plots show ΔStO_2_ stratified by grouped skin pigmentation (lighter pigmented: Fitzpatrick types 1–3; darker pigmented: Fitzpatrick type 5; Figure 2B). 

Lighter pigmented skin types showed positive ΔStO_2_ values (postoperative decrease), while darker pigmented skin showed negative values (postoperative increase). Boxes represent the interquartile range (IQR), horizontal lines indicate medians, whiskers extend to 1.5 × IQR, and points denote outliers.

## 4. Discussion

Various clinical methods have been implemented to improve early detection of ischemia and reduce postoperative complications related to MSF perfusion. Tissue oxygenation depends on microvascular perfusion and oxygen delivery, both of which are critical for flap survival. Impaired skin perfusion during mastectomy leads to hypoxia-driven metabolic changes, cellular injury, and ultimately necrosis if uncorrected [23,24]. Impaired perfusion causes hypoxia-driven metabolic injury and cell necrosis if uncorrected, underscoring the importance of early detection in reconstructive surgery to prevent irreversible tissue loss [23,24]. Studies have demonstrated that individuals with darker skin pigmentation are more likely to experience misdiagnosis of tissue ischemia when assessed through physical examination alone, highlighting the need for accurate and objective assessment tools [10]. Although numerous technologies have emerged to monitor postoperative tissue oxygenation, SnapshotNIR has successfully been used in the detection of oxygenation differences in chronic wounds [25,26,27], critical limb ischemia [28], and skin necrosis [29,30]. Additionally, its application in monitoring flap viability has increasingly shown promising results [21,30,31,32]. This handheld, lightweight device enables rapid and user-friendly operation, making it suitable for use in the operating room, hospital wards, and outpatient clinics [21]. Technologies that utilize surface detectors to measure tissue oxygenation, such as pulse oximeters, have also demonstrated racial bias, increasing the risk of undetected hypoxemia in individuals with darker skin pigmentation [16,19]. In this study, we evaluated the independent association between skin pigmentation, quantified using the Fitzpatrick scale, and changes in tissue oxygenation following mastectomy surgery measured with the SnapshotNIR device, a NIRS-based technology designed for real-time perfusion monitoring. First, we observed a significant negative correlation between Fitzpatrick score and ΔStO_2_, with higher Fitzpatrick scores demonstrating negative ΔStO_2_ values, suggesting an apparent postoperative increase in tissue oxygenation. Positive ΔStO_2_ values indicate reduced tissue oxygenation, whereas negative values indicate increased oxygenation relative to baseline, a distinction critical for accurate interpretation of these findings. Second, we confirmed that significant differences in ΔStO_2_ were demonstrated between Fitzpatrick scores 1 and 5, and between 2 and 5. These findings suggest that patients with darker skin pigmentation (e.g., Fitzpatrick 5) exhibit a false increase in StO_2_ measured postoperatively, in contrast to the expected decrease observed in lighter pigmented skin. This discrepancy raises concerns that NIRS-based devices may overestimate tissue oxygenation in darker skin pigmentation, particularly under hypoxemic conditions such as the mastectomy flap edges, thereby compromising measurement accuracy. While this may reflect underlying physiological variations in microvascular responses, it is more likely indicative of melanin-related optical absorption that may bias StO_2_ readings, potentially affecting the reliability of NIRS-based monitoring in patients with darker skin pigmentation [16,21]. NIRS technology has previously been widely validated for high sensitivity and specificity in detecting vascular compromise during flap monitoring [13]. However, factors influencing its reliability and accuracy remain inadequately explored [16,21,33]. Several studies have evaluated the impact of skin pigmentation on the accuracy of StO_2_ monitoring, consistently suggesting that, in darker skin pigmentation, changes in tissue oxygenation are systematically underestimated, potentially contributing to delayed recognition of tissue compromise [7,10,13,16,21,33].

In the context of flap monitoring, Westman et al. evaluated the ViOptix T.Ox device in a bicolor porcine flap model, allowing for direct comparison between pigmented and nonpigmented skin within the same flap. Following induced flap congestion and ischemia, StO_2_ changes were detectable in both skin types, but the magnitude was significantly more pronounced in nonpigmented skin [16]. Similarly, Polfer et al. examined the Vioptix NIRS probe in a human model of acute upper extremity ischemia induced by tourniquet insufflation. While StO_2_ decreased across all skin tones, the magnitude of change was also less pronounced in darker skin pigmentation [10]. These findings suggest that melanin-related measurement bias persists across different ischemia models and anatomical sites. The mechanism underlying these discrepancies is complex and remains to be fully elucidated. Melanin and hemoglobin both exhibit optical absorption within the near-infrared spectrum (700–1000 nm) [7,34,35,36], and melanin has been shown to impact the transmission of red and NIR light through the skin [7]. Additionally, increased melanin density at the skin surface reduces light penetration depth, contributing to the altered StO_2_ readings [7,34,35,36]. While these optical properties are known to influence signal accuracy, the exact pathways by which melanin affects NIRS-derived oxygenation measurements remain under investigation. Given the significant clinical implications of these findings, particularly in surgical settings where accurate real-time monitoring is crucial, clinicians should be made aware of the limitations of commercially available StO_2_ devices in patients with darker skin pigmentation regarding their underestimation of ischemia. Failure to recognize these biases may lead to false reassurance of tissue viability, increasing the risk of flap failure due to undetected ischemia.

To improve the reliability of the NIRS-based monitoring, future research should focus on refining device calibration [7]. Calibration methods could include multispectral corrections to separate hemoglobin and melanin signals and adaptive machine learning models trained on diverse skin pigmentations to adjust StO_2_ values for more accurate perfusion assessment [7]. This could involve modifying signal processing techniques or utilizing alternative wavelengths that penetrate deeper into the tissue [7,16,21,33,37]. Until this has been successfully established, multi-modal monitoring approaches, such as combining NIRS with hyperspectral imaging to provide spectral tissue oxygenation mapping or with Doppler ultrasound to assess real-time blood flow, may maintain diagnostic accuracy by integrating complementary perfusion and oxygenation data that are less reliant on optical absorption [7,38].

This study has several notable strengths. It employs a prospective quasi-experimental controlled trial design in which each patient serves as their own control, thereby minimizing inter-patient variability and accounting for potential confounders. While several studies have investigated the SnapshotNIR device, our study uniquely focuses on evaluating its performance across various Fitzpatrick skin types. In fact, this is the first study to examine the potential for racial bias in detecting tissue hypoperfusion using the SnapshotNIR device among individuals with darker skin pigmentation, as classified by the Fitzpatrick scale. These findings highlight the need for future refinements in device calibration and underscore the importance of accounting for skin pigmentation when evaluating the accuracy of technologies that use surface detectors. However, the authors acknowledge several limitations. Technical limitations include the device’s susceptibility to melanin-related optical absorption, which may bias StO_2_ readings and reduce accuracy in patients with darker skin pigmentation. Notably, melanin-related optical absorption remains relevant in ΔStO_2_ calculations, as prior studies indicate this effect is most pronounced during hypoxic states, which are central to perfusion assessment. Though intra-rater and inter-rater reliability was not formally evaluated in our study, we implemented measures to minimize variability and ensure consistency. For intra-rater reliability, all samples were analyzed in a single session by the same reviewer, which reduces potential inconsistencies associated with temporal or contextual differences. Regarding inter-rater reliability, we ensured the same reviewer applied identical methods to analyze both pre- and post-procedure images, thereby eliminating variability between different reviewers and maintaining consistent standards and criteria throughout the study.

While SnapshotNIR shows promise as a non-invasive tool for perfusion assessment, its current implementation may have limitations in providing accurate absolute perfusion values across all skin types, underscoring the need for further refinement and validation. Clinical limitations included the limited sample size, which may introduce bias, reduce statistical power to perform multivariable analysis for other patient’s factors, and restrict generalizability to a broader population. Moreover, the observed association between hormonal therapy and ΔStO_2_ is based on only two participants and should be interpreted cautiously. Only two participants represented Fitzpatrick types 1, 3, and 5, and none represented types 4 or 6, highlighting the need for broader inclusion across all skin types, especially darker pigmentation. Moreover, formal intra- and inter-rater reliability assessments were not performed; however, we mitigated this by analyzing all samples in a single session by the same reviewer using a standardized method to minimize variability. Additionally, the involvement of multiple surgeons with varying techniques and levels of experience may potentially influence outcomes limiting definitive conclusions. Future research with a larger, more diverse cohort and standardized protocols is necessary to validate and expand upon our findings, ideally including correlation of SnapshotNIR-derived StO_2_ measurements with gold-standard methods such as arterial blood gas analysis.

## 5. Conclusions

This study presents evidence demonstrating that skin pigmentation influences the accuracy of the SnapshotNIR, a near-infrared spectroscopy (NIRS)-based device, in monitoring changes in tissue oxygenation after mastectomy surgery. Skin types with a higher Fitzpatrick score are associated with an overestimation of tissue oxygen saturation, which may compromise the reliability of these measurements in clinical practice. The findings suggest that melanin absorption contributes to the observed inaccuracies, raising concerns regarding optical measurement bias that disproportionately affects individuals with darker skin. Therefore, advancements in device calibration and the development of correction algorithms accounting for skin pigmentation are urgently needed to improve the accuracy and clinical applicability of NIRS-based monitoring across diverse patient populations, and ongoing industry efforts demonstrate the feasibility of these improvements.

## Figures and Tables

**Table 1:** Fitzpatrick classification of skin types I through VI.

Fitzpatrick Skin Type	Description
**Type I**	White skin. Always burns, never tans.
**Type II**	Fair skin. Always burns, tans with difficulty.
**Type III**	Average skin color. Sometimes mild burn, tan about average.
**Type IV**	Light-brown skin. Rarely burns. Tans easily.
**Type V**	Brown skin. Never burns. Tans very easily.
**Type VI**	Black skin. Heavily pigmented. Never burns, tans very easily.

**Table 2:** Demographics and associations with ΔStO_2_. ΔStO_2_, change in tissue oxygenation calculated as preoperative StO_2_ minus postoperative StO_2_.

**ΔStO_2_ (Mean ± SD)**					
	**Y:N**	**%**	**Y**	**N**	***p*** **-value**
Smoking	7:26	21%	−1.5 ± 28.3	2.9 ± 21.7	0.658
Comorbidities	15:18	45%	0.8 ± 24.1	3.0 ± 22.3	0.787
Previous Breast Surgery	20:13	61%	3.4 ± 21.9	−0.2 ± 24.9	0.665
Adjuvant Therapy					
- Chemo	10:23	30%	4.3 ± 15.5	1.0 ± 25.6	0.711
- Hormonal	2:31	6%	37.9 ± 7.2	−0.3 ± 21.5	0.019
- Target	4:29	12%	−6.8 ± 7.9	3.2 ± 24.0	0.420
- Immunotherapy	1:32	3%	27.3 ± 0	1.2 ± 22.7	0.267
- (Any) Adjuvant	11:22	33%	7.8 ± 18.8	−0.9 ± 24.5	0.309
Complications	6:27	18%	0.1 ± 32.6	2.4 ± 4.0	0.831
**Correlation with ΔStO_2_**					
	**n**	**median**	**IQR**	ρ *	***p*-value**
Temperature	25	36.5	[36.1–36.7]	0.248	0.233
Systolic BP	30	119.5	[107–140]	0.139	0.465
Diastolic BP	30	75.0	[64–85]	0.355	0.054
Heart Rate	20	75.0	[64.5–83.3]	−0.130	0.586
Fitzpatrick Score	30	2	[2]	−0.086	0.650
* Spearman’s rho					
**Correlation with ΔStO_2_**					
	**n**	**mean**	**STdev**	ρ **	***p*-value**
Age	33	49.0	11.7	−0.125	0.489
** Pearson’s rho					

## Data Availability

The raw data generated and analyzed during the study are not publicly available as data sharing was not included in the informed consent. Deidentified data are available from the corresponding author upon reasonable request.

## Abbreviations

The following abbreviations are used in this manuscript:

StO_2_	tissue oxygenation
ΔStO_2_	change in tissue oxygenation, calculated as preoperative StO_2_ minus postoperative StO_2_
MSF	mastectomy skin flap
NIRS	near-infrared spectroscopy

## References

[1] Siegel R.L., Giaquinto A.N., Jemal A. (2024). Cancer statistics, 2024. CA Cancer J. Clin..

[2] Robertson S.A., Jeevaratnam J.A., Agrawal A., Cutress R.I. (2017). Mastectomy skin flap necrosis: Challenges and solutions. Breast Cancer Targets Ther..

[3] Sullivan S.R., Fletcher D.R., Isom C.D., Isik F.F. (2008). True incidence of all complications following immediate and delayed breast reconstruction. Plast. Reconstr. Surg..

[4] Mlodinow A.S., Fine N.A., Khavanin N., Kim J.Y. (2014). Risk factors for mastectomy flap necrosis following immediate tissue expander breast reconstruction. J. Plast. Surg. Hand Surg..

[5] Ito H., Ueno T., Suga H., Shiraishi T., Isaka H., Imi K., Miyamoto K., Tada M., Ishizaka Y., Imoto S. (2019). Risk factors for skin flap necrosis in breast cancer patients treated with mastectomy followed by immediate breast reconstruction. World J. Surg..

[6] Kohler L.H., Köhler H., Kohler S., Langer S., Nuwayhid R., Gockel I., Spindler N., Osterhoff G. (2021). Hyperspectral Imaging (HSI) as a new diagnostic tool in free flap monitoring for soft tissue reconstruction: A proof of concept study. BMC Surg..

[7] Pachyn E., Aumiller M., Freymüller C., Linek M., Volgger V., Buchner A., Rühm A., Sroka R. (2024). Investigation on the influence of the skin tone on hyperspectral imaging for free flap surgery. Sci. Rep..

[8] Olivier W.-A.M., Hazen A., Levine J.P., Soltanian H., Chung S., Gurtner G.C. (2003). Reliable assessment of skin flap viability using orthogonal polarization imaging. Plast. Reconstr. Surg..

[9] Lin S.J., Nguyen M.-D., Chen C., Colakoglu S., Curtis M.S., Tobias A.M., Lee B.T. (2011). Tissue oximetry monitoring in microsurgical breast reconstruction decreases flap loss and improves rate of flap salvage. Plast. Reconstr. Surg..

[10] Polfer E.M., Sabino J.M., Fleming I.C., Means K.R. (2019). Relative tissue oxygenation and temperature changes for detecting early upper extremity skin ischemia. Plast. Reconstr. Surg..

[11] Irwin M., Thorniley M., Dore C., Green C. (1995). Near infra-red spectroscopy: A non-invasive monitor of perfusion and oxygenation within the microcirculation of limbs and flaps. Br. J. Plast. Surg..

[12] Berthelot M., Ashcroft J., Boshier P., Hunter J., Henry F.P., Lo B., Yang G.-Z., Leff D. (2019). Use of near-infrared spectroscopy and implantable Doppler for postoperative monitoring of free tissue transfer for breast reconstruction: A systematic review and meta-analysis. Plast. Reconstr. Surg. Glob. Open.

[13] Kagaya Y., Miyamoto S. (2018). A systematic review of near-infrared spectroscopy in flap monitoring: Current basic and clinical evidence and prospects. J. Plast. Reconstr. Aesthetic Surg..

[14] Keller A. (2009). A new diagnostic algorithm for early prediction of vascular compromise in 208 microsurgical flaps using tissue oxygen saturation measurements. Ann. Plast. Surg..

[15] Wu C., Rwei A.Y., Lee J.Y., Ouyang W., Jacobson L., Shen H., Luan H., Xu Y., Park J.B., Kwak S.S. (2022). A wireless near-infrared spectroscopy device for flap monitoring: Proof of concept in a porcine musculocutaneous flap model. J. Reconstr. Microsurg..

[16] Westman A.M., Ribaudo J., Butler M., Shmuylovich L., Pet M.A. (2024). Skin Pigmentation Affects ViOptix T.Ox Performance in Variably Pigmented Preclinical Model of Flap Ischemia and Congestion. Plast. Reconstr. Surg. Glob. Open.

[17] Khavanin N., Yesantharao P., Kraenzlin F., Darrach H., Sacks J.M. (2021). Quantifying the effect of topical nitroglycerin on random pattern flap perfusion in a rodent model: An application of the ViOptix Intra. Ox for dynamic flap perfusion assessment and salvage. Plast. Reconstr. Surg..

[18] Khavanin N., Darrach H., Kraenzlin F., Yesantharao P.S., Sacks J.M. (2021). The Intra.Ox near-infrared spectrometer measures variations in flap oxygenation that correlate to flap necrosis in a preclinical rodent model. Plast. Reconstr. Surg..

[19] Sjoding M.W., Dickson R.P., Iwashyna T.J., Gay S.E., Valley T.S. (2020). Racial bias in pulse oximetry measurement. N. Engl. J. Med..

[20] Putcha A., Schichlein K., Nguyen T., Davis B., Choffin R., Malik S., Sharma A., Sosa G., Saffari S., Ribaudo J. (2025). Characterizing the influence of skin pigmentation on pulse oximetry. Biophotonics Discov..

[21] Moritz W.R., Daines J., Christensen J.M., Myckatyn T., Sacks J.M., Westman A.M. (2023). Point-of-care tissue oxygenation assessment with SnapshotNIR for alloplastic and autologous breast reconstruction. Plast. Reconstr. Surg. Glob. Open.

[22] Sachdeva S. (2009). Fitzpatrick skin typing: Applications in dermatology. Indian J. Dermatol. Venereol. Leprol..

[23] van den Heuvel M.G., Buurman W.A., Bast A., van der Hulst R.R. (2009). ischaemia–reperfusion injury in flap surgery. J. Plast. Reconstr. Aesthetic Surg..

[24] Rogoń I., Rogoń A., Kaczmarek M., Bujnowski A., Wtorek J., Lachowski F., Jankau J. (2024). Flap Monitoring Techniques: A Review. J. Clin. Med..

[25] Bowen R., Treadwell G., Goodwin M. (2016). Correlation of near infrared spectroscopy measurements of tissue oxygen saturation with transcutaneous pO2 in patients with chronic wounds. SM Vasc. Med..

[26] Serena T.E., Yaakov R., Serena L., Mayhugh T., Harrell K. (2020). Comparing near infrared spectroscopy and transcutaneous oxygen measurement in hard-to-heal wounds: A pilot study. J. Wound Care.

[27] Landsman A. (2020). Visualization of wound healing progression with near infrared spectroscopy: A retrospective study. Wounds.

[28] Gopalakrishnan S., Niezgoda J., Hoffman B., Siddique A., Niezgoda J.A. (2019). Using near infrared spectroscopy imaging to manage critical limb ischemia. Today’s Wound Clin..

[29] Jones G.E., Yoo A., King V.A., Sowa M., Pinson D.M. (2020). Snapshot multispectral imaging is not inferior to SPY laser fluorescence imaging when predicting murine flap necrosis. Plast. Reconstr. Surg..

[30] Hill W.F., Webb C., Monument M., McKinnon G., Hayward V., Temple-Oberle C. (2020). Intraoperative near-infrared spectroscopy correlates with skin flap necrosis: A prospective cohort study. Plast. Reconstr. Surg. Glob. Open.

[31] Takaya A., Tsuge I., Nakano T., Yamanaka H., Katsube M., Sakamoto M., Morimoto N. (2023). Flap viability evaluation using a tissue oximetry camera as an alternative to indocyanine green fluorescence imaging. Plast. Reconstr. Surg. Glob. Open.

[32] Chen Y., Shen Z., Shao Z., Yu P., Wu J. (2016). Free flap monitoring using near-infrared spectroscopy: A systemic review. Ann. Plast. Surg..

[33] Bai W., Guo H., Ouyang W., Weng Y., Wu C., Liu Y., Zang H., Jacobson L., Xu Y., Lu D. (2022). Intramuscular near-infrared spectroscopy for muscle flap monitoring in a porcine model. J. Reconstr. Microsurg..

[34] Rao R., Saint-Cyr M., Ma A.M.T., Bowling M., Hatef D.A., Andrews V., Xie X.-J., Zogakis T., Rohrich R. (2009). Prediction of post-operative necrosis after mastectomy: A pilot study utilizing optical diffusion imaging spectroscopy. World J. Surg. Oncol..

[35] Holmer A., Marotz J., Wahl P., Dau M., Kämmerer P.W. (2018). Hyperspectral imaging in perfusion and wound diagnostics–methods and algorithms for the determination of tissue parameters. Biomed. Eng. Biomed. Tech..

[36] Sood B.G., McLaughlin K., Cortez J. (2015). Near-infrared spectroscopy: Applications in neonates. Semin. Fetal Neonatal Med..

[37] Lu D., Li S., Yang Q., Arafa H.M., Xu Y., Yan Y., Ostojich D., Bai W., Guo H., Wu C. (2022). Implantable, wireless, self-fixing thermal sensors for continuous measurements of microvascular blood flow in flaps and organ grafts. Biosens. Bioelectron..

[38] Knoedler S., Hoch C.C., Huelsboemer L., Knoedler L., Stögner V.A., Pomahac B., Kauke-Navarro M., Colen D. (2023). Postoperative free flap monitoring in reconstructive surgery—Man or machine?. Front. Surg..

